# Downscaling SSP-consistent global spatial urban land projections from 1/8-degree to 1-km resolution 2000–2100

**DOI:** 10.1038/s41597-021-01052-0

**Published:** 2021-10-28

**Authors:** Jing Gao, Martino Pesaresi

**Affiliations:** 1 Department of Geography and Spatial Sciences, & Data Science Institute, University of Delaware, Newark, DE 19716 USA; 2 European Commission, Joint Research Centre, Directorate for Space, Security, and Migration, Ispra, I-21027 Italy

**Keywords:** Climate-change impacts, Socioeconomic scenarios, Environmental impact

## Abstract

Long-term, spatial urban land projections that simultaneously offer global coverage and local-scale empirical accuracy are rare. Recently a set of such projections was produced using data-science-based simulations and the Shared Socioeconomic Pathways (SSPs). These projections update at decadal time intervals from 2000 to 2100 with a spatial resolution of 1/8 degree, while many socio-environmental studies customarily run their analysis and modelling at finer spatial resolutions, e.g. 1-km. Here we develop and validate an algorithm to downscale the 1/8-degree spatial urban land projections to the 1-km resolution. The algorithm uses an iterative process to allocate the decadal amount of urban land expansion originally projected for each 1/8-degree grid to its constituent 1-km grids. The results are a set of global maps showing urban land fractions at the 1-km resolution, updated at decadal intervals from 2000 to 2100, under five different urban land expansion scenarios consistent with the SSPs. The data can support studies of potential interactions between future urbanization and environmental changes across spatial and temporal scales.

## Abstract (summary)

**Table:** 

Measurement(s)	urban land fraction
Technology Type(s)	computational downscaling • digital curation
Sample Characteristic - Environment	urban landscape
Sample Characteristic - Location	global

Machine-accessible metadata file describing the reported data: 10.6084/m9.figshare.16540080

## Background & Summary

Urban landscape is a primary venue of socio-environmental dynamics. For understanding the societal impacts of potential future urbanization and investigating its interactions with anticipated global environmental change over the 21st century, urban land projections that span long-term futures, are spatially-explicit, and offer global coverage with local-scale empirical accuracy, are necessary^1^. Though urbanization is known to be a local-scale process (different cities, even those in the same country, may expand in different styles) that changes over time, current global spatial urban land change models usually treat the world as less than twenty large regions and are based on snapshot data that remain static over time, neglecting significant spatiotemporal variations of the phenomenon. As a result, the credibility of current global spatial urban land projections^2–4^ is usually limited to near- or mid-term futures. Recently, a set of spatial urban land projections were developed that simultaneously meet all the above-listed requirements, using a new data-science-based modelling framework^5,6^, and urban land expansion scenarios consistent with the Shared Socioeconomic Pathways (SSPs)^7^.

Based on 15 best available global datasets on urbanization and its driving forces across spatial and temporal scales, this new modelling framework^5,6^ improved previous long-term urban land change modelling in three key aspects: (1) the new modelling framework captures changes in urbanization styles over time, and is especially equipped for making long-term projections; (2) the new modelling framework accounts for local-scale spatial variations in urbanization, by modelling the world as 375 sub-national regions (delineated according to locations of current settlement sites; more details in ref. ^2^) with one unique spatial model for each region; (3) with all its component models calibrated to spatially-explicit time-series data, the new modelling framework can identify the emergence of new urban centres, in contrast to previous urban models that generally expand existing urban centres for new land development. The new modelling framework was applied under five urban land expansion scenarios that are consistent with the SSPs^7^, resulting in a first set of empirically-grounded, global, spatial urban land projections throughout the 21st century. The dataset maps urban land fraction (a continuous variable ranging from 0 to 1) at the 1/8-degree resolution (which is roughly 14 km at the Equator), and updates at decadal time intervals from 2000 to 2100.

The 1/8-degree resolution is an ideal balance between capturing spatial details of urbanization and leaving out noisy signals at very fine spatial resolutions for long-term spatial urban land modelling^5^. 1/8-degree has been found more effective than other commonly-used spatial resolutions (including 1-km) for capturing empirical relationships between urban land expansion and its drivers (e.g. population change)^8^. However, many fields of socio-environmental analysis and modelling have customarily been tuned to finer spatial resolutions (e.g. 1-km) and would find the 1/8-degree resolution too coarse for their applications. To allow the recent advances in urban land change modelling to enable new analytical possibilities for more socio-environmental studies, here we develop and validate a downscaling algorithm, and use it to downscale the abovementioned 1/8-degree urban land projections^5^ to the 30-arc-second resolution (roughly 1-km) (below we denote this fine resolution as 1-km for notation simplicity). In the 1/8-degree as well as the 1-km datasets, “urban land” is defined as built-up land (a.k.a. impervious surface, or developed land), a land cover type signified by the presence of man-made materials and structures, e.g. cement, asphalt, buildings, roads, etc.

We tested alternative designs of the downscaling algorithm. The experiment descriptions and results are respectively presented in “Methods” and “Technical Validation”. We found the downscaling of urban land projections from 1/8-degree to 1-km can be reliably done using simple beginning-of-the-decade total urban land amount based spatial scalers. For a given decade, the downscaling algorithm allocates the 1/8-degree decadal amount of urban land expansion to 1-km grid cells in proportion to their total urban land amounts at the beginning of the decade. The algorithm uses an iterative process to collect any overflows from already highly developed 1-km grid cells, and allocate them to 1-km grid cells that are not yet fully developed within the same encompassing 1/8-degree grid cell. The iterative process repeats until all 1/8-degree amount of urban land expansion is allocated to 1-km grid cells with no overflow. The downscaling algorithm is applied decade by decade throughout the 21st century for every urban land expansion scenario.

The resulting dataset consists of global maps showing the fraction of urban land (a continuous variable ranging from 0 to 1) at the 1-km resolution, updated at decadal time intervals from 2000 to 2100, for five different urban land expansion scenarios consistent with the SSPs. Because the data span long-term futures, are spatially resolved at a fine resolution, and offer a global coverage with local-scale empirical accuracy, they can serve as key inputs to at least three types of socio-environmental studies involving urban land change: (1) global studies requiring a fine spatial resolution, (2) regional, local studies of long-term futures, and (3) integrated modelling fields that conventionally ignore urban land change (due to the longstanding lack of datasets like the one presented here), such as global long-term land use change, Earth system modelling, and land-atmosphere interactions^9^.

## Methods

The essence of downscaling urban land change over a given decade is to multiply the 1/8-degree decadal amount of urban land change by a 1-km spatial scaler to first produce a 1-km decadal urban land change amount map (Eq. (1)), and then generate the end-of-the-decade 1-km urban land fraction map accordingly. The decadal change is of focus here, because estimated change is the primary value of time-series urban land projections.

1 $$\Delta \;urban\;lan{d}_{1km}=\Delta \;urban\;lan{d}_{1/8degree}\times spatial\;scale{r}_{1km}$$

Different ways exist for deriving the 1-km spatial scaler, while an ideal spatial scaler for this application should meet two criteria: (1) focusing on the detailed spatial resolution range from 1/8 degree to 1 km and the short temporal interval of one decade, because the 1/8-degree projections have already accounted for primary urbanization trends and patterns at larger spatial and temporal scales with sophisticated modelling efforts, and (2) the simpler the better, not only because of the Occam’s razor principle of empirical modelling (i.e. the simplest model is usually the best one), but also because the complexity of the downscaling algorithm should match the amount of information to be handled, and the spatial resolution range from 1/8-degree to 1-km is only a secondary contributor to spatial variations in urban land expansion in comparison to the 1/8-degree and above scales.

For base data, we use the same global time-series urban land maps that the input 1/8-degree projections were based on, the Global Human Settlement Layer (GHSL)^10^. GHSL maps urban land (as built-up land) in a binary fashion (i.e. urban or not) at the 38-meter resolution for four time points – 1975, 1990, 2000, 2014. From GHSL, we extract the fraction of urban land within 1-km grid cells – a continuous variable ranging from 0 to 1 – and temporally linearly stretch the start- and the end-year 1-km urban land fraction maps to generate a regularly-spaced time series of 1980, 1990, 2000, and 2010.

### Alternative 1-km spatial scaler designs

Through empirical experiments, we tested the effectiveness of two alternative 1-km spatial scalers for downscaling decadal urban land expansion from 1/8-degree to 1-km. It is worth noting that this downscaling task is simpler than spatially modelling urban land change at the 1-km resolution from scratch, because the input 1/8-degree projections already accounted for important spatial and temporal variations of urban land change at the 1/8-degree and above scales. All our testing experiments and results should be interpreted in this context.

First, decadal change amount based spatial scaler:

2 $$spatial\;scale{r}_{1km}=\frac{\Delta \;urban\;lan{d}_{1km}}{\Delta \;urban\;lan{d}_{1/8degree}}$$

This is the exact definition of what is needed conceptually, however, the numerator Δ *urbanland*_1*km*_ does not exist before downscaling the denominator Δ *urbanland*_1/8*degree*_ for a given decade. We therefore test how the equation-(2)-based spatial scaler observed for one decade can be approximated by an equation-(2)-based spatial scaler from a previous decade or the average of spatial scalers from several decades. Using the GHSL-derived base data, we calculate equation-(2)-based spatial scalers for 1980–1990, 1990–2000, 2000–2010, and an average spatial scaler of the three decades; we then evaluate the pair-wise correlation among all these spatial scalers. If the scaler of one decade can be well approximated by a scaler from a previous decade or the average scaler, this method is a good candidate for our downscaling task.

Second, beginning-of-the-decade total amount based spatial scaler:

3 $$spatial\;scale{r}_{1km}=\frac{total\;urban\;lan{d}_{1km}}{total\;urban\;lan{d}_{1/8degree}}$$

We calculate the equation-(3)-based spatial scalers for 1980, 1990, and 2000, and evaluate how they respectively approximate the equation-(2)-based spatial scalers observed for the decadal urban land change over 1980–1990, 1990–2000, and 2000–2010. If the beginning-of-the-decade total amount based scaler is strongly correlated with their corresponding decadal change amount based scaler, this method is a good candidate for our downscaling task.

More complex spatial modelling is possible for generating the 1-km spatial scaler. However, since simplicity is an ideal characteristic for this downscaling effort, if either of the above two options works well, it is unnecessary to build more complex spatial models. As the results presented below in “Technical Validation” show, the beginning-of-the-decade total amount based spatial scaler performs well in terms of both accuracy and robustness. We therefore use equation-(3)-based spatial scalers in our downscaling algorithm, and applied it throughout the 21st century decade by decade for each of the five SSP-consistent urban land expansion scenarios.

### An iterative downscaling algorithm

When downscaling the 1/8-degree urban land projections throughout the 21st century, we update the equation-(3)-based spatial scaler at the beginning of each decade, use it in Eq. (1) to proportionally allocate the 1/8-degree amount of decadal urban land expansion to 1-km grid cells, and then repeat the process for the next decade. The equation-(3)-based spatial scaler allocates more new urban land expansion to 1-km grid cells with more existing urban land (than other 1-km grid cells within the same 1/8-degree grid cell). The simple proportional allocation could sometimes exceed the amount of available land remaining in some 1-km grid cells. Available land is the amount of developable land (i.e. total land area net of permanent ice and water^11^) that is not yet developed. We therefore use an iterative process that collects overflown allocations within each 1/8-degree grid cell and use Eq. (1) to proportionally allocate the total overflown amount to 1-km grid cells that still have land available for new development. As a result, 1-km grid cells with more existing urban land while still have available land will receive more overflown development (until they become fully developed), and the overflow from any 1-km grid cell does not go outside its encompassing 1/8-degree grid cell. For each decade, the iterative process repeats until all 1/8-degree grid cells’ amounts of urban land expansion is allocated to their respective constituent 1-km grid cells with no overflow. Because of the long time horizon and the wide range of the urban land expansion scenarios spanned by the input 1/8-degree urban land projections, and also because the modelling framework used to produce the 1/8-degree urban land projections is capable of identifying the emergence of new urban centres, it sometimes happens that a 1/8-degree grid cell’s amount of decadal urban land expansion is more than the total amount of remaining available lands from all 1-km grid cells that are somewhat developed at the beginning of the decade. The 1/8-degree grid cells still have available lands but they are in the 1-km grid cells that have no urban land at the beginning of the decade, which, according to Eq. (3), would have a zero weight in the 1-km spatial scaler. When this occurs for a 1/8-degree grid cell, our downscaling algorithm first fills up the 1-km grid cells that are somewhat developed at the beginning of the decade, and then allocates the remaining amount of the 1/8-degree grid cell’s urban land expansion to its other constituent 1-km grid cells in proportion to the 1-km grid cells’ amounts of available lands (Eq. (4)). The iterative allocation process is also applied here in case of any potential overflow.

4 $$spatial\;scale{r}_{1km}^{supplementary}=\frac{total\;available\;lan{d}_{1km}}{total\;available\;lan{d}_{1/8degree}}$$

**Fig. 1:**
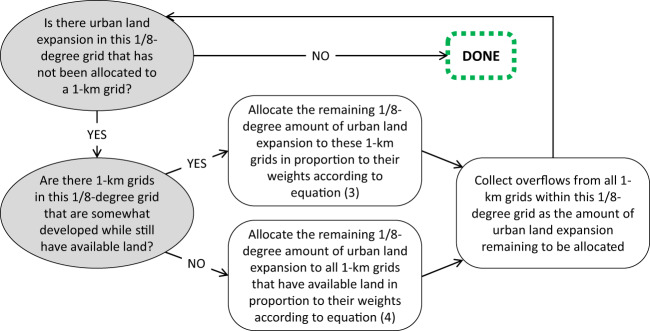
Conceptual workflow of the iterative downscaling algorithm for one 1/8-degree grid cell during one decade. The equation-(3)-based spatial scaler is updated at the beginning of every decade.

After downscaling the 1/8-degree decadal urban land expansion amount to the 1-km resolution (Fig. 1), we add the 1-km decadal change amount to the 1-km beginning-of-the-decade amount of urban land, resulting in the downscaled 1-km end-of-the-decade urban land amount. We then divide it by the total land area within each 1-km grid cell to produce the final product: 1-km fraction of urban land.

### Validations

Two sets of validations are conducted using the three decades (1980–2010) of observational data extracted from GHSL.

#### Short-term validation

For evaluating short-term performance, we use 2000–2010 as an example decade. We run the iterative downscaling algorithm to allocate the observed 1/8-degree amount of urban land expansion over the decade to the 1-km resolution, and contrast the downscaled 1-km amount of urban land expansion with the observed 1-km amount of urban land expansion. Because urban land expansion is often incremental at the 1-km resolution over short time horizons, we use a “null hypothesis” (i.e. assuming the observed 2000 urban land amount map is the estimated 2010 urban land amount map at the 1-km resolution) as the comparison baseline to offer a fair perspective for interpreting the downscaling algorithm’s performance level.

#### Mid-term validation

When downscaling the 1/8-degree urban land projections throughout the 21st century, it is reasonable to question how well the downscaling algorithm’s short-term performance level will hold for longer-term futures. Even though the downscaling algorithm updates the 1-km spatial scaler at the beginning of every decade, starting the second decade into the future the update is based on the downscaled results from the previous decade rather than actual observations at the 1-km resolution. To understand how the downscaling algorithm’s performance might change over time, we apply the downscaling process to the three decades with observational data (1980–2010) decade by decade consecutively for allocating the observed 1/8-degree decadal amounts of urban land expansion, and compare the spatial patterns of the downscaled 1-km urban land amount with the observed 1-km urban land amount at 1990, 2000, and 2010 (i.e., after applying the downscaling process for one decade, two decades, and three decades in a row). The less the performance level decays over time, the more confidence can be placed in the downscaling algorithm’s applicability for longer-term futures.

## Data Records

**Fig. 2:**
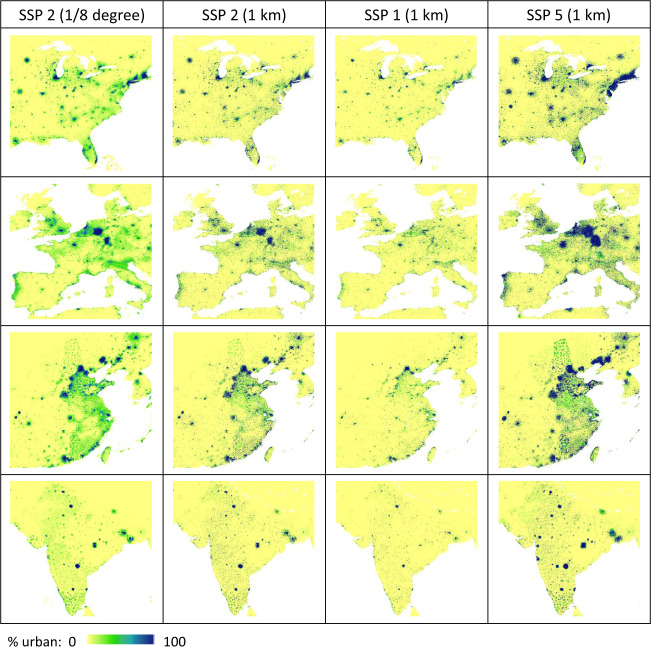
2100 urban land projections for various parts of the world (North America, Europe, East Asia, South Asia) under urban land expansion scenarios consistent with SSP 2 (middle of the road), SSP 1 (sustainability), and SSP 5 (fossil-fuelled development).

The dataset includes gridded global maps of urban land fractions for the period of 2000–2100 at the 1-km resolution and updates at decadal time intervals, under five urban land expansion scenarios consistent with the SSPs (SSP1 – sustainability, SSP2 – middle of the road, SSP3 – regional rivalry, SSP4 – inequality, SSP5 – fossil-fuelled development) (Fig. 2). The data are stored in two commonly-used file formats for geospatial data: GeoTIFF and NetCDF, and are publicly downloadable at 10.7927/1z4r-ez63^12^ (free registration and log-in may be needed according to requirements of the NASA Socioeconomic Data and Applications Center (SEDAC)). Each data file of future urban land projections is named as SSPx_yyyy, where x notes the SSP index and yyyy notes the year that the projection is made for. Two gridded global maps of ancillary data are also included: land_area_km^2^, which shows the total land area within each 1-km grid cell in the unit of km^2^ (multiplying this map with a projected urban land fraction map will result in a gridded map of urban land areas at the 1-km resolution); land_mask, which is a binary map showing all 1-km grid cells containing any land area. The input datasets to this study are available at 10.7910/DVN/ZHMI1L^5,13^ for the 1/8-degree SSP-consistent urban land projections (2000–2100), and 10.2905/jrc-ghsl-10007^10,14^ for the 1-km GHSL historical urban land observations (1975–2014).

## Technical Validation

### Comparison of alternative 1-km spatial scaler designs

As mentioned earlier, for approximating the conceptually exact but practically unattainable 1-km decadal change amount based spatial scaler, we tested the effectiveness of two alternative methods: (i) decadal change amount based 1-km spatial scalers from previous decades (e.g. how well the 2000–2010 change scaler can be approximated by the 1990–2000 change scaler, the 1980–1990 change scaler, or the 3-decade average scaler), and (ii) the beginning-of-the-decade total amount based 1-km spatial scaler (e.g. how well the 2000–2010 change scaler can be approximated by the 2000 total scaler). Table 1 shows the results of test (i), and Table 2 test (ii). All correlation scores in Table 1 are greater than 0.8, while the correlation scores in Table 2 are all greater than 0.9 and are very similar across different columns, indicating that the beginning-of-the-decade total amount based 1-km spatial scaler (Eq. (3)) performs more accurately as well as more robustly across different decades for this task. It therefore was selected as the basis of our downscaling algorithm.

**Table 1:** Correlation scores between 1-km spatial scalers based on observed change amounts over different decades.

	2000–2010 change scaler	1990–2000 change scaler	1980–1990 change scaler	3-scaler average
2000–2010 change scaler	1	0.91	0.84	0.85
1990–2000 change scaler	—	1	0.87	0.89
1980–1990 change scaler	—	—	1	0.96
3-scaler average	—	—	—	1

**Table 2:** Correlation scores between 1-km spatial scalers based on observed beginning-of-the-decade total amounts of urban land and those based on observed decadal change amounts, for the three decades with observational data.

	1980 total scaler vs. 1980–1990 change scaler	1990 total scaler vs. 1990–2000 change scaler	2000 total scaler vs. 2000–2010 change scaler
beginning-of-the-decade total amount scaler vs. decadal change amount scaler	0.94	0.91	0.92

### Short-term & Mid-term validations

**Fig. 3:**
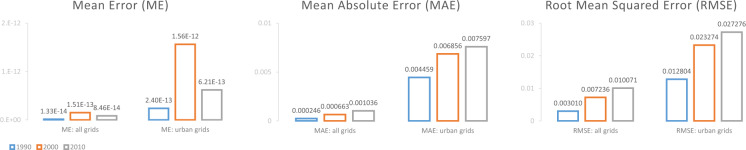
Mid-term validation: Error metrics comparing the downscaled and the observed 1-km decadal urban land expansion amounts (units: km^2^) for 1980–1990, 1990–2000, and 2000–2010, with 1980 being the beginning year, i.e. applying the downscaling algorithm consecutively for one decade, two decades, and three decades in a row.

When interpreting Table 3 and Fig. 3 validation results, we first focus on the mean absolute error (MAE) of urban grids – it shows the average magnitude of downscaling errors in urban areas, which prevents the vast amount of global lands that are not at all urban from diluting the reading of the metric. In the short-term validation (Table 3), our downscaling algorithm showed a noticeably lower average magnitude of error in urban areas (MAE: 0.003737 km^2^) than the null hypothesis (MAE: 0.006663 km^2^). In the mid-term validation (Fig. 3), the downscaling algorithm’s average magnitude of error in urban areas increases as the downscaling algorithm is applied consecutively for one decade (MAE: 0.004459 km^2^), two decades (MAE: 0.006856 km^2^), and three decades (MAE: 0.007597 km^2^), while the MAE at the end of the three-decade validation period is still low. As a benchmark for interpreting the magnitude of these errors, in the 2000 1-km observational data, the mean urban land area is 0.044605 km^2^ across urban grids and 0.003016 km^2^ across all land grids. These results instil confidence in that although the downscaling algorithm’s performance level decays as it is applied for long term, its average magnitude of error will likely remain reasonably low.

**Table 3:** Short-term validation: Error metrics comparing the downscaled and the observed 1-km decadal urban land expansion amounts (units: km^2^) for 2000–2010, with 2000 being the beginning year.

	Mean Error (ME)	Mean Absolute Error (MAE)	Root Mean Squared Error (RMSE)
all grids	−1.05E-13	0.000400	0.004350
urban grids	−9.80E-13	0.003737	0.013289
null-hypothesis:all grids	−0.000441	0.000441	0.004500
null-hypothesis: urban grids	−0.006663	0.006663	0.017486

The “null hypothesis” (assuming the observed 2000 urban land amount map is the estimated 2010 urban land amount map) provides a fair comparison baseline for interpreting the downscaling algorithm’s performance level.

**Fig. 4:**
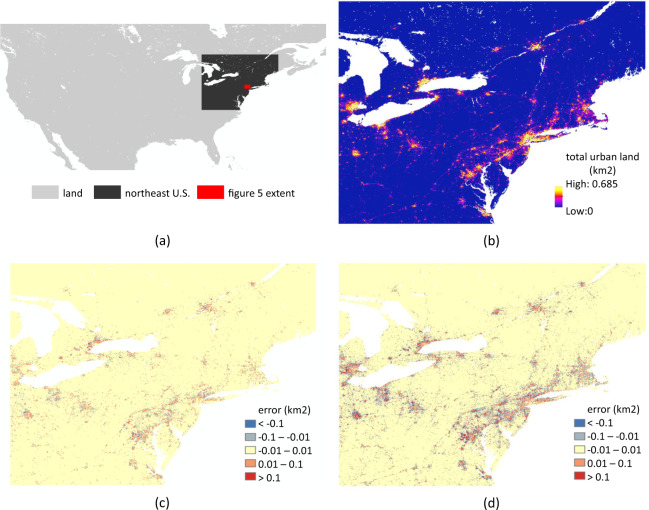
Validation results for one example metropolitan region (north-eastern U.S.): (**a**) location; (**b**) observed amount of urban land in 2010 (km^2^) at the 1-km resolution; (**c**) error in 2010 urban land amount (i.e. downscaled 1-km urban land amount – observed amount) (km^2^) from the short-term validation (applying the downscaling process for one decade); (**d**) error in 2010 urban land amount (km^2^) from the mid-term validation (applying the downscaling process for three decades in a row).

We also examined whether the downscaling algorithm’s errors show systematic biases. In Table 3 and Fig. 3, the mean errors (ME) of all validation experiments we run are near-zero, suggesting that the downscaling errors did not show systematic over- or under-estimation. Spatially, the downscaling algorithm’s over- and under-estimating errors show random spatial patterns across the world. For visual clarity, this point is illustrated in Fig. 4 with one example metropolitan region, since spatial details are difficult to see with small global maps, while similarly unbiased spatial randomness holds globally.

### Characteristics of the downscaling algorithm

**Fig. 5:**
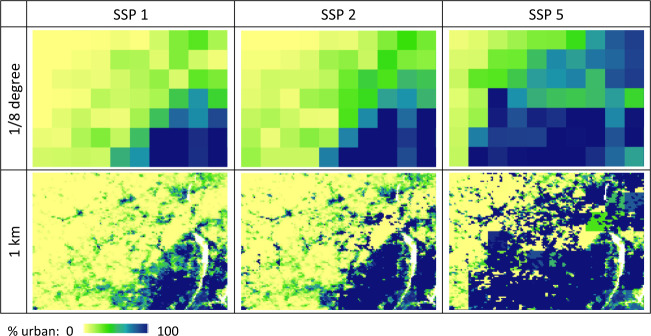
Characteristic patterns and possible artefacts of the downscaling algorithm: An example area (extent shown in Fig. 4a) illustrating the full range of %urban.

Despite the strong performance level the downscaling algorithm showed in validations, its properties create some characteristic patterns in the 1-km downscaled urban land projections (Fig. 5 and Fig. 2): (1) because the 1-km spatial scaler for allocating decadal change is based on the beginning-of-the-decade total amount of urban land, the downscaling algorithm fills up the already highly developed 1-km grid cells within each 1/8-degree grid cell first, as urbanization proceeds over time; (2) in the long-term future, especially when under a high urban land expansion scenario (e.g. SSP 5), some currently undeveloped or underdeveloped 1/8-degree grid cells can become quite developed, and because little variation at the 1-km resolution exists in historical data for these areas, their downscaled 1-km far-future projections may exhibit somewhat homogeneous blocky visual effects propagated from the 1/8-degree inputs.

## Usage Notes

The dataset presented here is a downscaled version (1-km) of a recently-published set of global long-term spatial urban land projections (1/8-degree). The new advances made and the challenges faced by the 1/8-degree urban land change modelling framework are therefore also embodied by the 1-km downscaled dataset. Carried over from the 1/8-degree projections, the 1-km downscaled dataset is also strong in projecting long-term futures, capturing local-scale spatial variations across the world, and identifying the emergence of new urban centres, while predicting detailed spatial patterns under a very high urban land expansion scenario (e.g. SSP 5) is also a challenge as mentioned above. Altogether, the 1-km downscaled dataset provides unprecedented empirically-grounded spatial precision for global long-term urban land projections.

We anticipate researchers working on three types of studies involving urban land change may find this dataset a useful input: (1) Global studies requiring a fine spatial resolution. (2) Regional, local studies of long-term futures. Although local-scale urban land change modelling often works at fine spatial resolutions, they are usually limited to near- or mid-term futures. Our work here fills this gap. By simultaneously maintaining local-scale empirical accuracy and a global coverage/consistency, this dataset also enables local-scale comparative studies of different parts of the world in the long-term future. (3) Integrated socio-environmental modelling and analyses that conventionally ignore urban land change due to the lack of global, long-term, spatial urban land projections like the dataset presented here, e.g. Earth system modelling, and studies of land-atmosphere interactions. Our work fills this gap and can enable investigations of urban-Earth-system interactions especially those of the global scale.

For urban land change modellers, spatially allocating decadal urban land expansion according to the beginning-of-the-decade total urban land amount might appear a counterintuitive choice. In fact, the study that produced this work’s input 1/8-degree urban land projections showed that calibration to time series of change rather than the initial total amount is essential for making accurate and robust mid- to long-term spatial urban land projections. Nonetheless, our empirical experiments here found that the beginning-of-the-decade total amount based spatial scaler outperforms the decadal change amount based spatial scaler, offering a more accurate and robust basis for the downscaling from 1/8-degree to 1-km. The difference in conclusions of the two studies is not surprising. It is because the 1/8-degree urban land projections have already accounted for key variations in urban land change at larger spatial and temporal scales, the downscaling from the 1/8-degree to the 1-km resolution can be reliably done using simple spatial scalers. The support that our empirical experiments provide for using the beginning-of-the-decade total amount based spatial scaler applies to the spatial downscaling from 1/8-degree to 1-km, but not modelling urban land change at the 1-km resolution in general.

Finally, our downscaled 1-km dataset has a few caveats worth noting: (1) The area of developable land is held constant across the projected scenarios and over time. Impacts of potential future environmental changes (e.g. sea level rise) are not incorporated. This is consistent with SSP-based projections (including the 1/8-degree dataset this downscaling work is based on). To enable applications of the dataset for studying interactions between urban land expansion and environmental change, such interactions cannot be endogenously built in the dataset. (2) Although urban land fractions are real numbers and are represented as floating-point values in this dataset, the precision of the 1-km urban fraction is the area of a 38-meter grid cell divided by the area of a 1-km grid cell, because the GHSL base data are binary (urban or not) at the 38-meter resolution. That is roughly $\frac{3{8}^{2}}{100{0}^{2}}=0.001444$, or three decimal digits. A related consideration is the minimal spatial support of the dataset (i.e. the size of the smallest patch of grid cells that can be reliably analysed). The consideration depends on what analyses the data are used for. For example, identifying comparative trends across scenarios for the same geographic area could mitigate methodological biases that are present in all scenarios (e.g. those of the downscaling algorithm), while quantifying the absolute spatial pattern would require more precision and accuracy. Naturally, the minimal spatial support of the dataset would be smaller for the former analysis than for the latter. (3) This dataset maps only the horizontal extent of urban land. Urban growth changing the vertical morphology of buildings (e.g. increasing the number of stories) is not explicitly considered, hence, grid cells with the same urban land fraction might have different population densities.

## Data Availability

The dataset was created using Python scripts with ArcGIS 10.6.1. Codes are publicly available at GitHub through Zenodo^15^. All input data are publicly available as described in “Data Records”.
